# Population age structure dependency of the excess mortality P-score

**DOI:** 10.1186/s12963-024-00346-w

**Published:** 2024-09-27

**Authors:** Niklas Ullrich-Kniffka, Jonas Schöley

**Affiliations:** 1 University of Rostock, Ulmenstr. 69, 18057 Rostock, Germany; 2 Max Planck Institute for Demographic Research, Konrad-Zuse-Str. 1, 18057 Rostock, Germany

**Keywords:** Excess mortality, Decomposition, COVID-19, P-score, Standardization

## Abstract

**Background:**

Since the outbreak of the COVID-19 pandemic, the excess mortality P-score has gained prominence as a measure of pandemic burden. The P-score indicates the percentage by which observed deaths deviate from expected deaths. As the P-score is regularly used to compare excess mortality between countries, questions arise regarding the age dependency of the measure. In this paper we present formal and empirical results on the population structure bias of the P-score with a special focus on cross-country comparisons during the COVID-19 pandemic in Europe.

**Methods:**

P-scores were calculated for European countries for 2021, 2022, and 2023 using data from the 2024 revision of the United Nations’ World Population Prospects and the HMDs Short Term Mortality Fluctuations data series. The expected deaths for 2021, 2022, and 2023 were estimated using a Lee–Carter forecast model assuming pre-pandemic conditions. P-score differences between countries were decomposed using a Kitagawa-type decomposition into excess-mortality and structural components. To investigate the sensitivity of P-score cross-country rankings to differences in population structure we calculated the rank-correlation between age-standardized and classical P-scores.

**Results:**

The P-score is an average of age-specific percent excess deaths weighted by the age-distribution of expected deaths. It can be shown that the effect of differences in the distribution of deaths only plays a marginal role in a European comparison. In most cases, the excess mortality effect is the dominant effect. P-score rankings among European countries during the COVID-19 pandemic are similar under both age-standardized and classical P-scores.

**Conclusions:**

Although the P-score formally depends on the age-distribution of expected deaths, this structural component only plays a minor role in a European comparison, as the distribution of deaths across the continent is similar. Thus, the P-score is suitable as a measure of excess mortality in a European comparison, as it mainly reflects the differences in excess mortality. However, this finding should not be extrapolated to global comparisons, where countries could have very different death distributions. In situations were P-score comparisons are biased age-standardization can be applied as a solution.

## Introduction

The P-score is a widely used measure for excess mortality and has received great attention since the outbreak of the COVID-19 pandemic. It indicates the percentage difference between the observed and expected number of deaths in a population over some time interval [1–3]. Expected deaths result from an arbitrary counterfactual scenario. The P-score has been widely used to estimate levels of excess mortality during the COVID-19 pandemic [1, 2, 4–16]. Cross-country comparisons of P-scores are potentially problematic because P-scores are susceptible to structural differences of populations [17]. The consensus seems to be that P-scores depend on the age structure of a population and that the P-score favors younger populations over older ones, resulting in lower P-scores for younger populations [9, 11, 18, 19]. Given that the COVID-19 pandemic disproportionately affected the elderly [19], and that P-scores are often used to compare the effectiveness of different countries’ pandemic responses, it is important to recognize that population structure can introduce bias into these comparisons: Two populations may show different total P-scores despite having identical age-specific P-scores.

The issue of population structure biases in ratios of observed to expected counts has been discussed in the context of the standardized mortality ratio (SMR). As early as 1934, Yule recognized that aggregate mortality ratios are an average of age-specific mortality ratios weighted by the expected mortality distribution over population strata [20]. Because the weights are different for different populations, SMR comparisons between populations can be misleading [21]. To avoid bias when comparing mortality ratios between populations, a standard mortality distribution should be used for weighting [20, 22]. We demonstrate and expand these earlier results in the context of the P-score during the COVID-19 pandemic in Europe. As the P-score is closely related to the SMR our results apply equally to indirectly standardized ratios of counts.

In this paper we formally and empirically analyze the age-dependence of the P-score. First we derive the P-score as an average of age-specific percent excess deaths weighted by the expected distribution of deaths over age. Using this expression we analyze the age-structure bias of the P-score under proportional and linear changes in age-specific percent excess deaths. We contrast the P-score, a death weighted average, with the per capita number of excess deaths, a population weighted average, and show that both measure are closely related via the crude death rate. Based on the death weighted average we propose a Kitagawa-type [23] decomposition of P-score differences into into an age-specific excess mortality effect and an expected distribution of deaths effect. We apply this decomposition to P-score differences among European countries during the years 2021, 2022, and 2023. Finally, we calculate standardized P-scores that are insensitive to differences in the expected distribution of deaths and contrast them to classical P-scores to determine the sensitivity of P-score country rankings to population structure biases.

## The age dependency of the P-score

The P-score *P* is defined as

1 $$\begin{aligned} P = \frac{D^{\textrm{O}} - D^{\textrm{E}}}{D^{\textrm{E}}} \end{aligned}$$

where $D^O$ denotes the actual deaths observed in a population over a period of time and $D^E$ denotes the number of expected deaths resulting from an arbitrary counterfactual scenario [1–3]. Often this counterfactual scenario relates to a situation where an alternative set of mortality rates is acting on the population. Thus, the P-score is a measure that indicates the percentage difference between observed deaths and expected deaths. It has a co-domain from − 1 to infinity, where − 1 means that 100% fewer than expected deaths were observed, i.e. no one died, while any higher value can be interpreted as a percentage. The measure is undefined in the case of zero expected deaths.

The total number of observed and expected deaths over a period of time can be expressed as the sum of the age-specific number of deaths over the same period

2 $$\begin{aligned} P = \frac{\sum _x D_x^O - \sum _x D_x^E}{\sum _x D_x^E} = \frac{\sum _x D_x^O - D_x^E}{\sum _x D_x^E} \end{aligned}$$

with index *x* referring to age(-groups). The age-specific number of deaths on the other hand is the result of the age-specific mortality rate $m_x$ acting on the populations exposure $N_x$

3 $$\begin{aligned} P = \frac{\sum _x m_x^ON_x - m_x^EN_x}{\sum _x m_x^EN_x} \end{aligned}$$

with superscript *O* denoting the observed mortality rate and *E* denoting expected mortality rate, respectively. The observed age-specific mortality rates can be expressed as the expected age-specific mortality rates, scaled by the rate-ratio $\gamma _x$

4 $$\begin{aligned} P = \frac{\sum _x \gamma _x m_x^EN_x - m_x^EN_x}{\sum _x m_x^EN_x} = \frac{\sum _x (\gamma _x - 1) m_x^EN_x}{\sum _x m_x^EN_x}. \end{aligned}$$

Since $\gamma _x$ is the ratio of observed to expected deaths, both of which are positive values, subtracting 1 (and multiplying with 100) gives the percentage difference from observed to expected deaths which we denote by $\varphi _x = \gamma _x-1$. The P-score can then be expressed as the weighted sum

5 $$\begin{aligned} P = \sum _x \varphi _x \frac{m^E_x N_x}{\sum _x m_x^E N_x} = \sum _x \varphi _x \frac{D^E_x}{D^E} = \sum _x \varphi _x \pi _x, \end{aligned}$$

with $\pi _x$ the age-specific share of expected death to all expected deaths, i.e. the expected age distribution of deaths. This shows that the P-score depends on both the age-specific percentage difference of observed mortality to the expected mortality and the expected age distribution of death. The total P-score is, in fact, an average of age-specific P-scores weighted by the age distribution of people who would have died under the expected rates. The expected distribution of deaths was found by Yule [20] as a weight for the standardized mortality ratio (SMR), and since the P-score and the SMR are closely related, it should not be surprising that this weight also applies to the P-score.

We can further show that attempts to express the total P-score as an average of age-specific P-scores weighted by age-specific population exposures, lead back to the death-weighted average of Eq. (5). Rewriting the number of deaths in Eq. (5) as a product of mortality rates and the populations exposure one has

6 $$\begin{aligned} P = \sum _x \varphi _x \frac{D^E_x}{D^E} = \sum _x \varphi _x \frac{m^E_x N_x}{CDR^E N} = \sum _x \varphi _x \frac{m^E_x}{CDR^E}\frac{N_x}{N} \end{aligned}$$

with $CDR^E$ the average expected death rate of the population, better know as crude death rate [24]. Mortality rates can be expressed by the ratio of deaths over populations exposure

7 $$\begin{aligned} P = \sum _x \varphi _x \frac{D_x^E / N_x}{D^E / N}\frac{N_x}{N} = \sum _x \varphi _x \frac{D_x^E}{N_x}\frac{N}{D^E}\frac{N_x}{N} = \sum _x \varphi _x \frac{D_x^E}{D^E}\left[ \frac{N}{N_x}\frac{N_x}{N}\right] . \end{aligned}$$

Equation (7) shows that the total P-score is therefore not a pure function of age-specific P-scores and population exposure proportions but rather a pure function of age-specific P-scores and proportions of expected deaths.

To obtain a measure of excess mortality that actually depends on the age structure of the living population, the P-score (Eq. 6) can be multiplied by the expected crude death rate. This yields

8 $$\begin{aligned} P \cdot CDR^E = \sum _x \varphi _x m_x^E \frac{N_x}{N} = \sum _x \frac{(m_x^O - m_x^E)m_x^E}{m_x^E} \frac{N_x}{N} = \sum _x (m_x^O - m_x^E) \frac{N_x}{N} = RD_{p.c.} \end{aligned}$$

which is identical to the excess deaths per capita denoted as $RD_{p.c.}$ (risk difference per capita) [2].

### An age-constant change in death rates

Equation (5) can be used to explore the dynamics of the P-score given different functions for $\varphi _x$ and $\pi _x$. Generally, the P-score depends on both the age-specific P-score and the expected distribution of death. But there is one exception, namely when expected death rates are elevated by the same factor along the age-range, formally when $\varphi _x = \varphi$ one has

9 $$\begin{aligned} P = \sum _x \varphi _x \pi _x = \sum _x \varphi \,\pi _x = \varphi \sum _x \pi _x = \varphi , \end{aligned}$$

as the death proportions sum to 1 over age. Therefore, in a *proportional hazards* scenario, where expected mortality is elevated by a constant factor over age, the age-specific excess factor can be estimated from total counts of observed and expected deaths alone. In any other case the expected distribution of deaths over age is influencing the total P-score.

### An age-linear change in death rates

Assume for example that the age-specific P-scores changes linear with age, formally $\varphi _x = a+bx$. In this case the P-score can be expressed as

10 $$\begin{aligned} P = \sum _x (a+bx)\pi _x \end{aligned}$$

with *a* the intercept and *b* the slope. Dissolving parentheses yields

11 $$\begin{aligned} P = \sum _x a\,\pi _x + bx\,\pi _x = \sum _x a\,\pi _x + \sum _x bx\,\pi _x. \end{aligned}$$

Since *a* and *b* are constants, these parameters can be pulled out of the sum.

12 $$\begin{aligned} P = a\sum _x \pi _x + b\sum _x x\,\pi _x \end{aligned}$$

The first summation sign resembles the distribution of death over the whole age range, which is 1. The second summation sign is the age-weighted distribution, which is the expected mean age at death in a population ${\overline{x}}^E$

13 $$\begin{aligned} P = a + b {\overline{x}}^E. \end{aligned}$$

Clearly, under the scenario of a linear change in mortality with age, the P-score depends on the expected structure of deaths in a population. If the slope of $\varphi _x$ is positive, meaning older ages experience a higher proportional increase in death rates over expected, then the higher the average age at death in the population under the expected scenario, the higher the P-score. In other words, for two populations with equal intercept and $b > 0$, the population with a higher mean age at death will have a higher P-score. The opposite holds for $b < 0$.

### A decomposition of the P-score

In reality, $\varphi _x$ will most certainly not be constant, linear or follow any simple parametric progression over age. Equation (5) resembles the case with an arbitrary shape for $\varphi _x$ and $\pi _x$. Therefore populations with equal P-score can have varying $\varphi _x$ and/or $\pi _x$. To find the influence of either the shape of the age-specific P-scores and the distribution of expected deaths, we can decompose the difference between two total P-scores, $P^B$ and $P^A$, using Kitagawa’s decomposition approach [23]. This approach isolates the difference of the age-specific P-scores and the difference in expected density.

14 $$\begin{aligned} \Delta P = P^B - P^A = \underbrace{\sum _x (\varphi ^B_x - \varphi ^A_x)\left( \frac{\pi ^A_x + \pi ^B_x}{2}\right) }_{\varphi -\textrm{effect}} + \underbrace{\sum _x (\pi ^B_x - \pi ^A_x)\left( \frac{\varphi ^A_x + \varphi ^B_x}{2}\right) }_{\pi -\textrm{effect}} \end{aligned}$$

The first sum resembles the $\varphi$-effect, which indicates how much of the difference between two P-scores is attributable to differences in age-specific P-scores between population *A* and *B*. The latter summand indicates the effect size resulting from differences between the expected mortality distributions, namely the $\pi$-effect. The sign of each effect indicates whether it contributed to a decrease or increase to the P-score difference. The effect sizes can also be expressed as a percentage of the total effect. To do this, the absolute values of each effect is set in relation to the sum of the absolute values of all effects. The formula for the $\%\varphi$-effect is as follows

15 $$\begin{aligned} \%{\varphi \mathrm {-effect}} = \frac{|\varphi \mathrm {-effect}|}{|\varphi \mathrm {-effect}| + |\pi \mathrm {-effect}|}. \end{aligned}$$

Analogously, the formula for the $\%\pi$-effect looks like this:

16 $$\begin{aligned} \%{\pi \mathrm {-effect}} = \frac{|\pi \mathrm {-effect}|}{|\varphi \mathrm {-effect}| + |\pi \mathrm {-effect}|}. \end{aligned}$$

Both Eqs. (15) and (16) have a co-domain between 0 and 1, but both of them must add up to 1. 0 means 0% influence and 1 means 100% influence, respectively.

## Decomposition of P-score differences

We calculate the contribution of structural differences to cross-country differences between P-scores in 2021, 2022 and 2023 across Europe. The decomposition equation (14) requires the number of observed and expected deaths by country, sex and age.

Observed deaths were sourced from the Short Term Mortality Fluctuation Data Series (STMF) [25]. STMF data comes in a weekly format with abridged age groups. We aggregated the weeks to annual data and ungrouped the abridged death counts using the penalized composite link model [26, 27], a non-parametric disaggregation method for histograms of count data, resulting in single age groups from 0 to 100+. We choose to include European countries in our analysis if more than 10 age groups and at least 52 calendar weeks of data were available within a year. After adjustment, the data still includes the following countries: Austria, Belgium, Bulgaria, Croatia, the Czech Republic, Denmark, Estonia, Finland, France, Germany, Hungary, Iceland, Italy, Latvia, Lithuania, Luxembourg, the Netherlands, Norway, Poland, Portugal, Slovakia, Slovenia, Spain, Sweden and Switzerland.

The number of expected deaths was estimated using the Lee–Carter model [28] under pre-pandemic conditions. As this model extrapolates mortality rates we used midyear population counts from the World Population Prospects (WPP) [29] as exposures for the death counts. We forecasted mortality rates by age, sex, and country for 2021, 2022 and 2023 based on pre-pandemic trends. The baseline period for the forecast was 2000 to 2019, except for Italy (2011–2019), Denmark (2007–2019), and the Czech Republic (2005–2019). The resulting expected mortality rates were then converted to expected number of deaths by multiplying with the exposures. P-scores could then be calculated using the observed number of deaths and the number of deaths estimated under pre-pandemic assumptions. The associated predictions intervals were derived by sampling from the predictive distribution of the forecasted mortality rates using the StMoMo package [30].

Differences between two P-scores were decomposed according to Eq. (14) and the relative impact of the age-specific excess mortality effect $\varphi$-effect was calculated according to Eq. (15).

Table 1 shows the P-scores in percent and the corresponding 95% confidence interval for all included countries, separately for women and men in 2021. Most P-scores are significantly different from 0, except for women and men in Iceland and Luxembourg.

In 2021, we observe the highest P-score in Bulgaria, recording 41.32% more deaths among women and 42.79% among men compared to expected figures. The lowest P-score among included countries (excluding non significant P-scores) was − 2.36% for women in Austria and 2.48% for men in Norway. The median P-score was 6.93% for women and 11.00% for men in 2021.

**Table 1:** P-scores in 2021 for women and men for all included European countries

Country	Female		Male	
	P-score (%)	95% CI	P-score (%)	95% CI
Austria	− 2.36	(− 3.24; − 1.46)	8.24	(7.20; 9.30)
Belgium	− 1.66	(− 2.46; − 0.83)	6.96	(6.07; 7.88)
Bulgaria	41.32	(40.10; 42.56)	42.79	(41.60; 44.00)
Switzerland	1.70	(0.64; 2.77)	6.06	(4.94; 7.22)
Czechia	20.21	(19.22; 21.22)	29.12	(28.07; 30.20)
Germany	4.07	(3.78; 4.36)	8.21	(7.90; 8.52)
Denmark	5.66	(4.40; 6.96)	5.22	(4.00; 6.48)
Spain	6.37	(5.91; 6.83)	9.10	(8.64; 9.57)
Estonia	17.28	(14.81; 19.86)	20.64	(17.94; 23.46)
Finland	4.89	(3.66; 6.15)	5.65	(4.42; 6.91)
France	4.15	(3.79; 4.53)	7.73	(7.35; 8.12)
Croatia	18.72	(17.31; 20.16)	21.01	(19.56; 22.51)
Hungary	16.96	(16.08; 17.86)	24.12	(23.15; 25.11)
Iceland	*0.01*	(− 5.44; 6.06)	*− 3.12*	(− 8.24; 2.62)
Italy	6.93	(6.57; 7.29)	10.97	(10.59; 11.37)
Lithuania	25.84	(24.11; 27.64)	23.63	(21.88; 25.44)
Luxembourg	*2.01*	(− 2.13; 6.45)	*3.72*	(− 0.48; 8.16)
Latvia	26.06	(24.05; 28.13)	26.48	(24.32; 28.71)
Netherlands	9.15	(8.39; 9.93)	13.87	(13.07; 14.70)
Norway	3.62	(2.23; 5.06)	2.48	(1.09; 3.92)
Poland	24.25	(23.71; 24.79)	27.97	(27.43; 28.52)
Portugal	11.11	(10.19; 12.05)	11.75	(10.83; 12.69)
Slovakia	37.17	(35.52; 38.87)	38.03	(36.43; 39.69)
Slovenia	7.19	(5.18; 9.25)	16.31	(14.09; 18.62)
Sweden	0.97	(0.05; 1.92)	4.55	(3.59; 5.54)

Italic numbers indicate P-scores are not significantly different from zero. Source: World Population Prospects (2024) and Short Term Mortality Fluctuation data series (2024)

The P-scores show how heterogeneously the various countries were affected by the pandemic in 2021. Nevertheless, the question arises to what extent these results are comparable with each other, as the P-score depends not only on age-specific excess mortality but also on a structural component, namely the expected distribution of deaths. The results of the P-score decomposition are shown below. Here, differences between two P-scores were divided into the excess mortality effect and the expected mortality distribution effect. These absolute effects were then converted into percentage effect sizes.

**Fig. 1:**
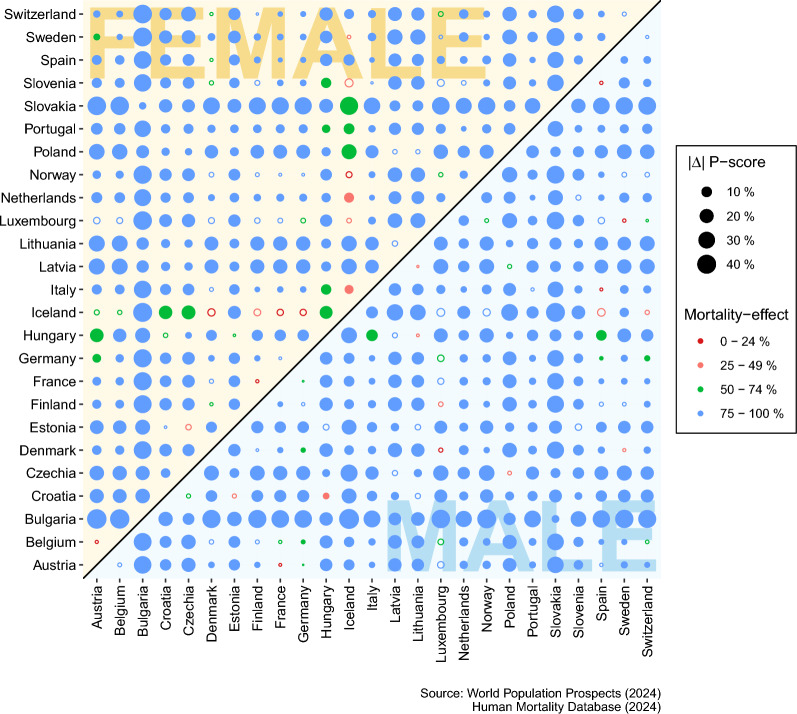
Absolute P-score differences and excess mortality effect strength in percent. Each point represents the absolute P-score difference between two countries in 2021. The larger the dot, the greater the absolute difference between the P-scores. A filled dot indicates a significant difference, while a hollow dot indicates a nonsignificant difference. The color of the dots indicates the strength of the excess mortality effect. The higher the percentage, the greater the influence of the excess mortality effect. The yellow area shows the results for women and the blue area shows the results for men. Source: World Population Prospects (2024), Human Mortality Database (2024)

Figure 1 shows the absolute P-score differences $\Delta$ between countries and the corresponding influence of the excess mortality effect. The size of each dot indicates the absolute difference between two P-scores. A filled dot indicates a significant difference between two P-scores, whereas a hollow circle indicates a non-significant difference between two P-scores. There is a non-significant difference if the confidence intervals overlap. The color of each dot indicates the impact of the excess mortality effect ($\varphi$-effect). A blue dot means that the excess mortality effect explains between 75% and 100% of the difference, while a green dot means that between 50% and 74% is explained by the excess mortality effect. An orange dot means that the excess mortality effect explains between 25% and 49% of the difference and a red dot means that the excess mortality effect explains up to 24%. In the last two cases, the structural effect is the dominant effect. The yellow area shows the results for women and the blue area shows the results for men. The diagram reveals a compelling insight: in most instances, 75–100% of the differences between P-scores can be attributed to the excess mortality effect. There are, however, notable exceptions. In a few cases, the effect of excess mortality is only 50–74%, especially if one of the countries of decomposition is Iceland. In most cases where the structural effect is the dominant effect, the difference between two P-scores is either small or not significant.

Similar results can be seen for 2022 and 2023, although the pattern of excess mortality has changed over the years. The supplementary Table 1 shows the P-scores for all included countries in 2022. P-scores for Luxembourg are not significantly different from 0. It can be seen that excess mortality decreased in Eastern European countries compared to 2021, while excess deaths increased in many other European countries. The lowest observed P-score was − 4.06% for women in Austria and 6.20% for men in Sweden. The highest observed P-score in 2022 was 15.14% for women in Finland and 16.16% for men in Bulgaria. The median P-score was 8.27% for women and 9.28% for men. The supplementary Fig. 1 shows the impact of the excess mortality effect on the absolute P-score differences between countries for 2022. Most significant differences can be explained mainly by the excess mortality effect.

In 2023, excess mortality decreased in every included European country compared with 2022 (see supplementary Table 2). The P-scores for women from Switzerland, Iceland, Lithuania, Luxembourg and Latvia are not significantly different from 0, as are the P-scores for men from Bulgaria, the Czech Republic, Iceland, Lithuania and Slovakia. The highest P-score in 2023 was observed for Finnish women with a value of 9.79% and for Finnish men with 12.45%. The lowest P-score was observed for women in Austria (− 10.95%) and for men in Luxembourg (− 4.67%). Absolute P-score differences were mainly explained by the excess mortality effect in 2023, as can be seen in the supplementary Fig. 2.

The results show that the excess mortality effect is the explanatory factor in most cases and that differences in mortality structure play a minor role. At first glance, therefore, P-scores appear to be well suited for comparing countries.

## Age standardized P-score

As demonstrated, the structural component has minimal impact on the absolute difference between two P-scores. This is likely because our analysis only included European countries, which have relatively similar expected death distributions. In less developed countries with lower life expectancies and higher mortality rates in younger age groups, the expected death distribution differs, leading to a potentially more significant structural component in such comparisons. However, even if the structural component has a minor influence, the influence is not zero. To make two P-scores even more comparable, age standardization can be performed. This means that the same distribution of deaths is assumed for two populations [20, 22]. This type of standardization allows the difference between two P-scores to be reduced to the excess mortality component. Theoretically, any structure can be chosen, but a reasonable one should be chosen. For example, one population may have a higher mortality effect than another population in every age group except the first. If we now create a standard mortality structure with 100% deaths in the first age group, we get a different result than if we only had no deaths in the first age group. There is no universally valid procedure for choosing the standard mortality distribution, because effects, i.e. changes in the direction of the differences, are possible with any type of turnover. We chose a combined standard distribution of all included countries stratified by age, sex and year. Formally we adjust Eq. (5) by changing the distribution of deaths to a standard distribution $\pi _{x,st.}$

17 $$\begin{aligned} P_{st.} = \sum _x \varphi _x \pi _{x,st.} \end{aligned}$$

Table 2 shows the P-score, the standardized P-score and their respective ranking among the included countries, as well as the difference between the standardized and the empirical P-score separately for women and men in 2021. Looking at the last column for women and men, we see that the P-score changes very little after adjustment. With the exception of Icelandic women, for whom the P-score has changed by a full 9.61% points after standardization. The P-score for Icelandic women is not significant due to a small number of deaths, which is why the expected distribution of deaths is not robust and can lead to biased results. The other P-score differences vary between − 5.48% points and 2.67% points. This relatively low variability due to standardization can also be seen in the ranking of P-scores and standardized P-scores. Only in a few cases did the rank change as a result of standardization. Spearman’s Rho confirms this impression with a value of 0.938 for women and 0.992 for men, both significant.

**Table 2:** P-scores, standardized P-scores, the difference between P-score and the standardized P-score ($\Delta$P-score) and their respective rank (#) in 2021 for men and women for all included European countries

Country	Female					Male				
	P-score	#	$\text {P-score}_{st.}$	#	$\Delta$P-score	P-score	#	$\text {P-score}_{st.}$	#	$\Delta$P-score
Austria	− 2.36	1	0.31	2	2.67	8.24	11	10.11	11	1.87
Belgium	− 1.66	2	− 0.45	1	1.21	6.96	8	7.96	8	1.00
Bulgaria	41.32	25	35.84	25	− 5.48	42.79	25	41.97	25	− 0.82
Switzerland	1.67	5	2.00	4	0.33	5.95	7	6.99	7	1.04
Czechia	20.21	20	19.13	20	− 1.08	29.12	23	27.98	22	− 1.14
Germany	4.07	8	4.26	7	0.19	8.21	10	8.92	10	0.71
Denmark	5.66	11	5.57	10	− 0.09	5.22	5	4.95	3	− 0.27
Spain	6.37	12	7.83	12	1.46	9.10	12	10.33	12	1.23
Estonia	17.28	18	18.04	18	0.76	20.64	17	20.78	18	0.14
Finland	4.89	10	5.47	9	0.58	5.65	6	6.35	5	0.70
France	4.15	9	4.68	8	0.53	7.73	9	8.64	9	0.91
Croatia	18.72	19	18.48	19	− 0.24	21.01	18	20.50	17	− 0.51
Hungary	16.95	17	14.35	17	− 2.60	24.11	20	22.07	19	− 2.04
Iceland	0.01	3	9.62	15	9.61	− 3.12	1	− 1.22	1	1.90
Italy	6.93	13	7.28	11	0.35	10.97	13	12.13	14	1.16
Lithuania	25.84	22	25.38	23	− 0.46	23.63	19	24.37	20	0.74
Luxembourg	2.01	6	2.75	5	0.74	3.72	3	6.61	6	2.89
Latvia	26.06	23	25.30	22	− 0.76	26.48	21	25.53	21	− 0.95
Netherlands	9.15	15	9.56	14	0.41	13.87	15	14.25	15	0.38
Norway	3.62	7	3.87	6	0.25	2.48	2	2.96	2	0.48
Poland	24.25	21	24.25	21	0.00	27.97	22	29.33	23	1.36
Portugal	11.11	16	11.52	16	0.41	11.75	14	11.98	13	0.23
Slovakia	37.17	24	34.59	24	− 2.58	38.03	24	36.79	24	− 1.24
Slovenia	7.19	14	7.99	13	0.80	16.31	16	16.68	16	0.37
Sweden	0.97	4	1.37	3	0.40	4.55	4	5.56	4	1.01

Standardization was done using a combined distribution of deaths. *Source*: World Population Prospects (2024), Short Term Mortality Fluctuation data series (2024)

For 2022 and 2023 we find similar results. Supplementary Table 3 shows the standardization for 2022, and after standardization Iceland has the highest P-score increase from 14.28 to 18.85%, which is an increase of 4.57% points. The other P-score differences after standardization range from − 1.31 percentage points to 2.83% points. Spearman’s Rho is 0.980 for women and 0.920 for men in 2022, indicating a high rank correlation. The supplementary Table 4 shows the standardization results for 2023. Iceland’s P-score increases by 8.63% points after standardization. Again, we have the case that many of the decomposition results with Icelandic women are not significant and in most cases the excess mortality effect is below 75% (see supplementary Fig. 2). This makes Iceland more sensitive to standardization. Other P-scores change between − 1.19% points and 2.97% points after standardization. Again, we observe a high Spearman’s Rho of about 0.945 for women and 0.962 for men, indicating a high rank correlation.

If a uniform density is used, i.e. the same proportion dies in each age group, there are clear differences in rank, as can be seen in supplementary Tables 5 to 7. In these cases Spearman’s Rho ranges from 0.2 to 0.539. However, a uniform density is an extreme distribution that is not very realistic and only serves to illustrate the importance of choosing an appropriate standard population.

## Discussion

The P-score is a measure that was widely used during the COVID-19 pandemic to map pandemic events and quantify excess mortality. It is a measure of the percentage difference between observed and expected mortality. Formally, it can be shown that the P-score is not an age-standardized measure, nor does it represent the sum of age-specific P-scores weighted by the age-structure of a population. Still, the P-score is not free of structural influences. The P-score is a sum of age-specific P-scores weighted by the expected distribution of deaths [20, 22].

Nevertheless, any structural influence is a possible source of bias. Populations with the same age-specific P-score may have different P-scores due to different expected mortality structures. To uncover a possible bias, we decomposed the P-scores for selected European countries for 2021, 2022, and 2023 into the influence of different age-specific P-scores and the influence of different mortality distributions using a Kitagawa-like decomposition method [23].

Cross-European comparisons show that P-score comparisons are robust and are not influenced much by the structural effect. In almost all cases, actual differences in age-specific P-scores are the key factors for total P-score differences. Even standardization with a combined mortality structure of included countries stratified by year and sex only minimally changes the ranking of P-scores and standardized P-scores. Spearman’s Rho underlines this correlations with values of over 0.938 for women and over 0.920 men in 2021 to 2023.

We found that although the P-score is not an age-standardized measure, it is robust to differences in population age structure across Europe. Other factors, such as the choice of baseline model, are much more consequential for rankings in cross-country excess death comparisons [3]. However, this finding should not be extrapolated to global comparisons. In a European comparison, death structures are very similar, so this component loses significance. In global comparisons, where countries have very different death distributions, it may well be that the P-score is not a good measure of excess mortality. In such a case, standardization may be useful. Further research in this area is needed to see how much the P-score is biased by expected death distributions in countries with very different death distributions. Large-scale or global comparisons [9, 31] may be biased here.

In addition, the results should not be extrapolated to other mortality events, such as heat waves or influenza epidemics, because other dynamics may be present. Some simplifications were made to calculate P-scores for the different countries. For example, the same population was assumed for actual and expected deaths. Furthermore, the P-scores were calculated at an annual level, which means that intra-year seasonal effects were not taken into account and considerable variations within a year are possible.

The P-score is closely related to other measures of excess mortality, first and foremost, the Standardized Mortality Ratio. Both are relative measures of excess counts, and neither are age-standardized but instead depend on the expected age distribution of deaths. Scaling the P-score with the total number of expected deaths per capita gives the risk difference per capita, or excess death rate, an additive measure of excess mortality that is likewise sensitive to structural influences. However, the structural influence in this case is not the expected mortality structure, but the age structure of the living population.

## Supplementary Information

Additional file 1.

## Data Availability

(1) The population exposures were provided in the 2024 revision of the World Population Prospects by the United Nations. URL: https://population.un.org/wpp/Download/Standard/CSV/. (2) The observed death counts were provided by the Short Term Mortality Fluctuation data series, which is a part of the Human Mortality Database (mortality.org) and are openly accessible under: https://www.mortality.org/Data/STMF.
