# Supplementary material for: Population age structure dependency of the excess mortality P-score
Source: Popul Health Metr. 2024 Sep 27;22:25. doi: 10.1186/s12963-024-00346-w (PMC11428885; doi:10.1186/s12963-024-00346-w)
Supplement: Supplementary file 1 — Additional file 1. [file 12963_2024_346_MOESM1_ESM.pdf]

6 Supplementary Files

Country	Female		Male	
	P-score (%)	95% CI	P-score (%)	95% CI
Austria	-4.06	(-4.91; -3.19)	8.40	(7.37; 9.44)
Belgium	3.15	(2.31; 4)	6.72	(5.82; 7.63)
Bulgaria	14.41	(13.42; 15.42)	16.16	(15.18; 17.16)
Switzerland	5.82	(4.73; 6.92)	8.79	(7.64; 9.98)
Czechia	6.03	(5.15; 6.92)	7.65	(6.77; 8.54)
Germany	7.46	(7.16; 7.76)	10.36	(10.05; 10.68)
Denmark	9.93	(8.62; 11.27)	8.89	(7.62; 10.19)
Spain	10.59	(10.12; 11.08)	10.34	(9.87; 10.81)
Estonia	7.01	(4.78; 9.32)	11.85	(9.34; 14.47)
Finland	15.14	(13.79; 16.51)	14.75	(13.42; 16.12)
France	7.37	(6.99; 7.75)	9.01	(8.63; 9.41)
Croatia	8.68	(7.4; 9.99)	6.76	(5.49; 8.07)
Hungary	3.85	(3.07; 4.64)	8.02	(7.18; 8.89)
Iceland	14.28	(8.05; 21.13)	7.50	(1.87; 13.72)
Italy	8.27	(7.91; 8.64)	9.28	(8.89; 9.66)
Lithuania	13.54	(11.99; 15.16)	10.15	(8.6; 11.77)
Luxembourg	*0.76*	(-3.33; 5.16)	*-0.04*	(-3.99; 4.23)
Latvia	10.34	(8.58; 12.18)	12.62	(10.7; 14.62)
Netherlands	10.13	(9.37; 10.91)	10.49	(9.7; 11.28)
Norway	11.21	(9.72; 12.76)	13.01	(11.48; 14.6)
Poland	5.78	(5.33; 6.24)	8.00	(7.54; 8.46)
Portugal	12.53	(11.59; 13.48)	10.60	(9.69; 11.53)
Slovakia	10.77	(9.44; 12.13)	11.97	(10.67; 13.31)
Slovenia	5.83	(3.88; 7.9)	9.28	(7.2; 11.44)
Sweden	4.48	(3.52; 5.45)	6.20	(5.24; 7.2)

Supplementary Table 1: P-scores in 2022 for women and men for all included European countries. *Italic* numbers indicate P-scores are not significantly different from zero. Source: World Population Prospects (2024) and Short Term Mortality Fluctuation data series (2024)

Country	Female		Male	
	P-score (%)	95% CI	P-score (%)	95% CI
Austria	-10.95	(-11.72; -10.16)	4.63	(3.65; 5.64)
Belgium	-3.52	(-4.3; -2.74)	2.14	(1.28; 3.01)
Bulgaria	-2.02	(-2.87; -1.14)	*-0.62*	(-1.46; 0.24)
Switzerland	*0.65*	(-0.37; 1.7)	3.06	(1.98; 4.17)
Czechia	-2.77	(-3.56; -1.95)	*0.45*	(-0.36; 1.27)
Germany	2.23	(1.95; 2.52)	5.44	(5.15; 5.74)
Denmark	6.16	(4.9; 7.47)	6.94	(5.7; 8.21)
Spain	2.92	(2.49; 3.37)	3.50	(3.06; 3.94)
Estonia	-3.28	(-5.29; -1.2)	3.87	(1.57; 6.28)
Finland	9.79	(8.51; 11.1)	12.45	(11.14; 13.79)
France	1.23	(0.87; 1.59)	3.07	(2.71; 3.45)
Croatia	-4.11	(-5.24; -2.95)	-3.22	(-4.38; -2.03)
Hungary	-3.90	(-4.61; -3.17)	1.35	(0.56; 2.16)
Iceland	*2.05*	(-3.47; 8.21)	*5.11*	(-0.36; 11.06)
Italy	-0.97	(-1.3; -0.63)	1.48	(1.12; 1.83)
Lithuania	*-0.88*	(-2.24; 0.54)	*0.25*	(-1.17; 1.73)
Luxembourg	*0.48*	(-3.53; 4.78)	-4.67	(-8.42; -0.59)
Latvia	*-1.45*	(-3.01; 0.18)	4.11	(2.34; 5.95)
Netherlands	9.03	(8.27; 9.8)	10.09	(9.32; 10.89)
Norway	6.74	(5.3; 8.23)	8.03	(6.56; 9.53)
Poland	-5.90	(-6.3; -5.5)	-3.47	(-3.87; -3.06)
Portugal	5.60	(4.74; 6.49)	6.00	(5.13; 6.89)
Slovakia	-1.89	(-3.05; -0.68)	*0.09*	(-1.06; 1.28)
Slovenia	-2.70	(-4.49; -0.84)	6.26	(4.25; 8.35)
Sweden	2.95	(2.02; 3.91)	5.05	(4.09; 6.03)

Supplementary Table 2: P-scores in 2023 for women and men for all included European countries. *Italic* numbers indicate P-scores are not significantly different from zero. Source: World Population Prospects (2024) and Short Term Mortality Fluctuation data series (2024)

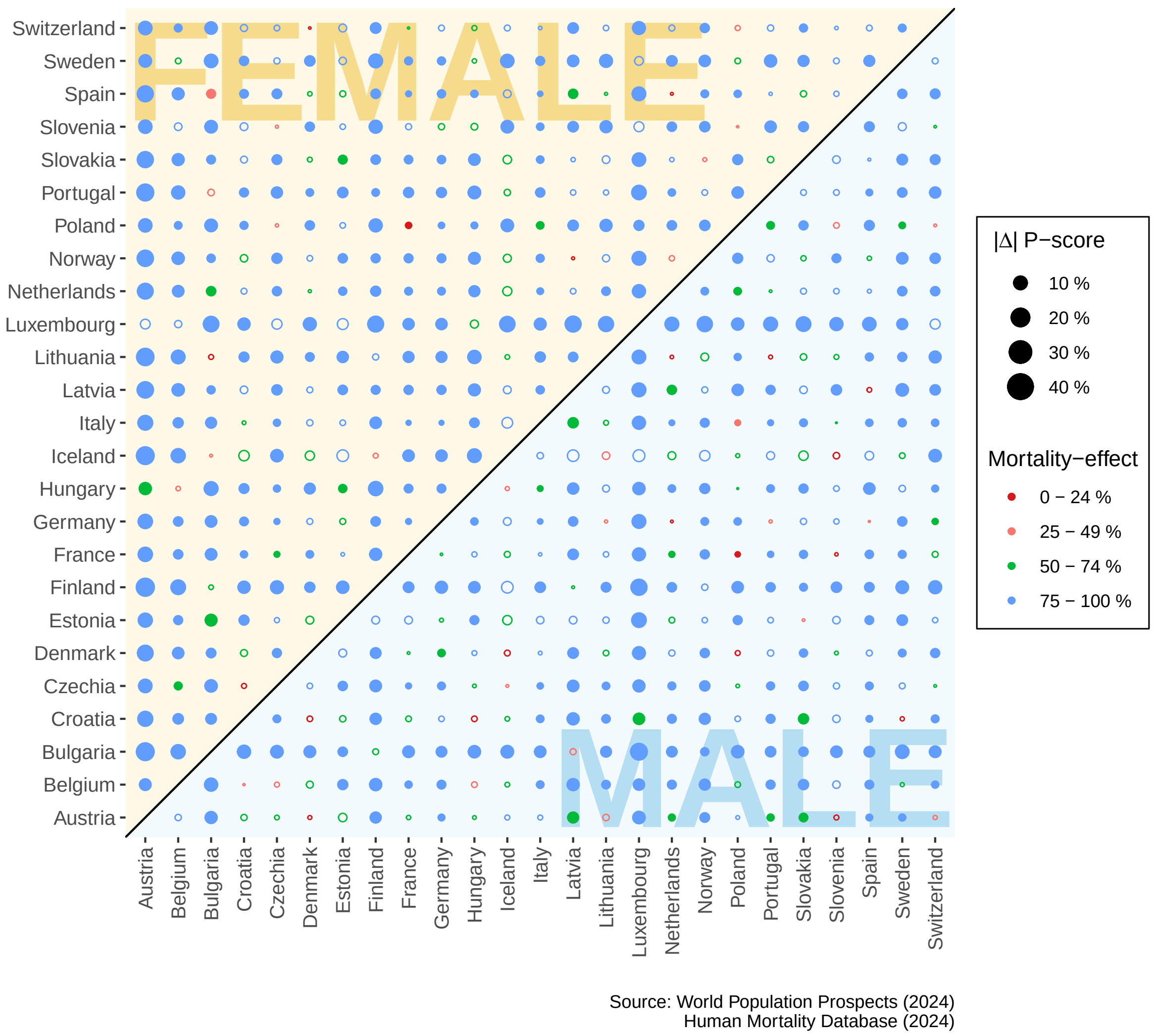

Supplementary Figure 1: Absolute P-score differences and excess mortality effect strength in percent. Each point represents the absolute P-score difference between two countries in 2022. The larger the dot, the greater the absolute difference between the P-scores. A filled dot indicates a significant difference, while a hollow dot indicates a nonsignificant difference. The color of the dots indicates the strength of the excess mortality effect. The higher the percentage, the greater the influence of the excess mortality effect. The yellow area shows the results for women and the blue area shows the results for men. Source: World Population Prospects (2024), Human Mortality Database (2024)

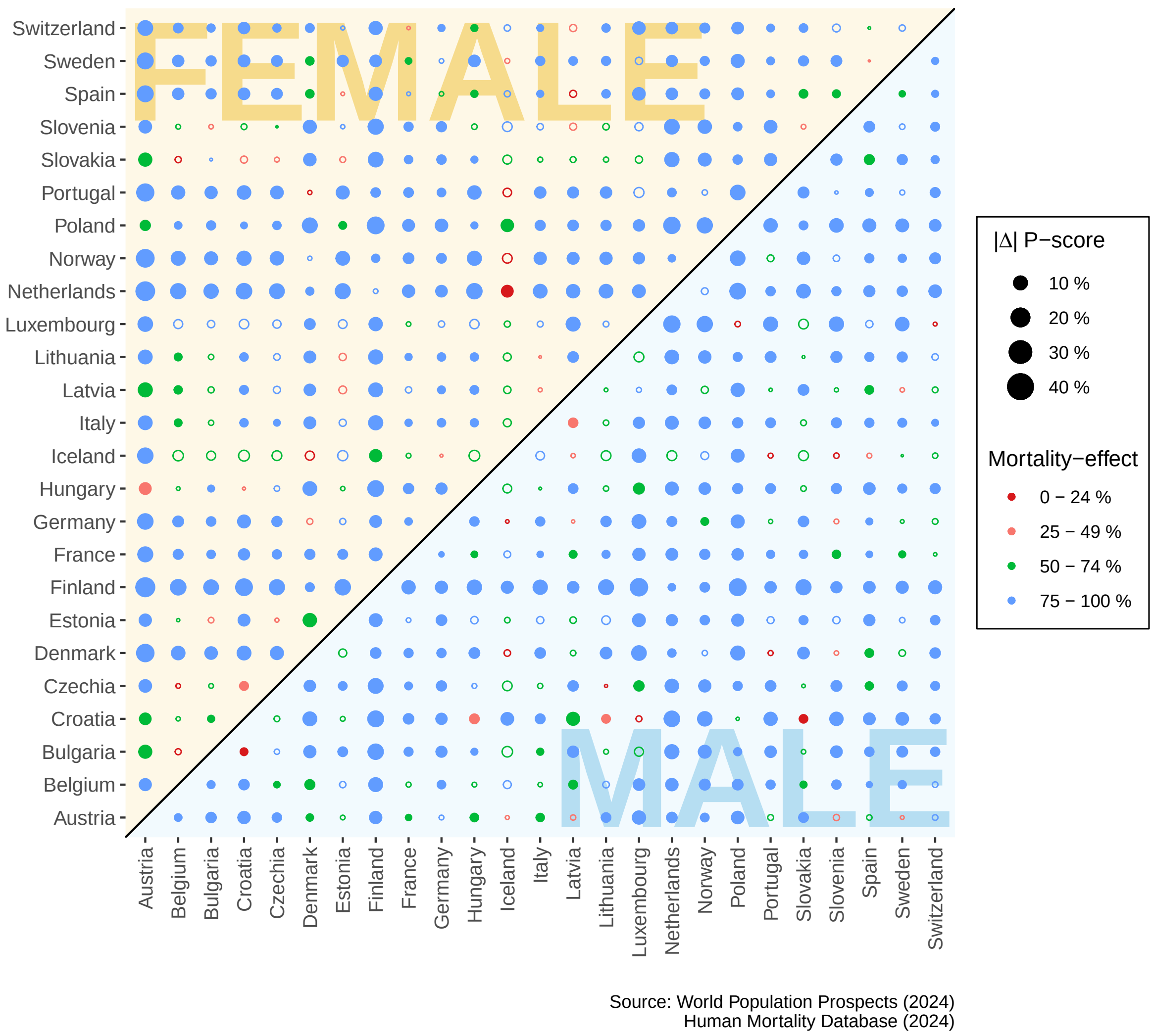

Supplementary Figure 2: Absolute P-score differences and excess mortality effect strength in percent. Each point represents the absolute P-score difference between two countries in 2023. The larger the dot, the greater the absolute difference between the P-scores. A filled dot indicates a significant difference, while a hollow dot indicates a nonsignificant difference. The color of the dots indicates the strength of the excess mortality effect. The higher the percentage, the greater the influence of the excess mortality effect. The yellow area shows the results for women and the blue area shows the results for men. Source: World Population Prospects (2024), Human Mortality Database (2024)

Country	Female					Male				
	P-score	#	P-score$_{st.}$	#	ΔP-score	P-score	#	P-score$_{st.}$	#	ΔP-score
Austria	-4.06	1	-1.23	1	2.83	8.40	9	10.00	14	1.60
Belgium	3.15	3	4.28	4	1.13	6.72	3	7.43	4	0.71
Bulgaria	14.41	24	13.10	22	-1.31	16.16	25	17.19	25	1.03
Switzerland	5.92	8	6.01	6	0.09	8.96	11	8.97	8	0.01
Czechia	6.03	9	6.57	7	0.54	7.65	6	7.60	5	-0.05
Germany	7.46	12	7.60	11	0.14	10.36	17	11.03	17	0.67
Denmark	9.93	15	9.54	15	-0.39	8.89	10	8.74	7	-0.15
Spain	10.59	17	11.40	18	0.81	10.34	16	11.22	18	0.88
Estonia	7.01	10	7.98	13	0.97	11.85	20	11.98	20	0.13
Finland	15.14	25	15.87	24	0.73	14.75	24	15.47	24	0.72
France	7.37	11	7.49	10	0.12	9.01	12	9.56	11	0.55
Croatia	8.68	14	9.11	14	0.43	6.76	4	6.97	3	0.21
Hungary	3.85	4	3.30	3	-0.55	8.02	8	7.67	6	-0.35
Iceland	14.28	23	18.85	25	4.57	7.50	5	9.81	12	2.31
Italy	8.27	13	7.96	12	-0.31	9.28	13	9.36	9	0.08
Lithuania	13.54	22	13.66	23	0.12	10.15	15	11.22	19	1.07
Luxembourg	0.76	2	1.96	2	1.20	-0.04	1	1.57	1	1.61
Latvia	11.38	20	11.43	19	0.05	14.60	23	14.28	23	-0.32
Netherlands	10.13	16	10.57	16	0.44	10.49	18	11.02	16	0.53
Norway	11.21	19	11.28	17	0.07	13.01	22	12.78	22	-0.23
Poland	5.78	6	7.14	9	1.36	8.00	7	9.86	13	1.86
Portugal	12.53	21	12.68	21	0.15	10.60	19	10.81	15	0.21
Slovakia	10.77	18	11.82	20	1.05	11.97	21	12.23	21	0.26
Slovenia	5.83	7	6.60	8	0.77	9.28	14	9.45	10	0.17
Sweden	4.48	5	4.73	5	0.25	6.20	2	6.23	2	0.03

Supplementary Table 3: P-scores, standardized P-scores, the difference between P-score and the standardized P-score (ΔP-score) and their respective rank (#) in 2022 for men and women for all included European countries. Standardization was done using a combined distribution of deaths. Source: World Population Prospects (2024), Short Term Mortality Fluctuation data series (2024)

Country	Female					Male				
	P-score	#	P-score$_{st.}$	#	ΔP-score	P-score	#	P-score$_{st.}$	#	ΔP-score
Austria	-10.95	1	-7.98	1	2.97	4.63	15	6.10	17	1.47
Belgium	-3.52	5	-2.26	8	1.26	2.14	10	2.91	10	0.77
Bulgaria	-2.02	9	-2.82	6	-0.80	-0.62	4	-1.11	4	-0.49
Switzerland	0.92	15	1.13	15	0.21	3.39	12	3.56	11	0.17
Czechia	-2.77	7	-2.42	7	0.35	0.45	7	0.12	6	-0.33
Germany	2.23	18	2.74	17	0.51	5.44	18	6.56	20	1.12
Denmark	6.16	22	5.50	20	-0.66	6.94	22	6.21	18	-0.73
Spain	2.92	19	4.65	19	1.73	3.50	13	5.10	15	1.60
Estonia	-3.28	6	-1.19	10	2.09	3.87	14	4.65	14	0.78
Finland	9.79	25	10.59	24	0.80	12.45	25	13.04	25	0.59
France	1.23	16	1.52	16	0.29	3.07	11	3.95	12	0.88
Croatia	-4.11	3	-3.98	4	0.13	-3.22	3	-2.43	2	0.79
Hungary	-3.91	4	-5.05	2	-1.14	1.33	8	0.50	8	-0.83
Iceland	2.05	17	10.68	25	8.63	5.11	17	7.79	22	2.68
Italy	-0.97	11	-0.75	11	0.22	1.48	9	2.27	9	0.79
Lithuania	-0.88	12	-0.74	12	0.14	0.25	6	0.38	7	0.13
Luxembourg	0.48	14	0.80	14	0.32	-4.67	1	-2.53	1	2.14
Latvia	-0.46	13	-0.63	13	-0.17	5.71	19	4.58	13	-1.13
Netherlands	9.03	24	9.32	23	0.29	10.09	24	10.62	24	0.53
Norway	6.75	23	6.93	22	0.18	8.05	23	8.17	23	0.12
Poland	-5.90	2	-5.03	3	0.87	-3.47	2	-2.26	3	1.21
Portugal	5.60	21	6.07	21	0.47	6.00	20	6.64	21	0.64
Slovakia	-1.89	10	-3.08	5	-1.19	0.09	5	-0.87	5	-0.96
Slovenia	-2.70	8	-1.65	9	1.05	6.26	21	6.37	19	0.11
Sweden	2.95	20	3.22	18	0.27	5.05	16	5.33	16	0.28

Supplementary Table 4: P-scores, standardized P-scores, the difference between P-score and the standardized P-score (ΔP-score) and their respective rank (#) in 2023 for men and women for all included European countries. Standardization was done using a combined distribution of deaths. Source: World Population Prospects (2024), Short Term Mortality Fluctuation data series (2024)

Country	Female					Male				
	P-score	#	P-score$_{st.}$	#	ΔP-score	P-score	#	P-score$_{st.}$	#	ΔP-score
Austria	-2.36	1	-2.03	1	0.33	8.24	11	7.00	5	-1.24
Belgium	-1.66	2	2.67	3	4.33	6.96	8	7.92	9	0.96
Bulgaria	41.32	25	37.79	24	-3.53	42.79	25	32.96	23	-9.83
Switzerland	1.67	5	6.42	7	4.75	5.95	7	7.91	8	1.96
Czechia	20.21	20	14.37	13	-5.84	29.12	23	17.84	16	-11.28
Germany	4.07	8	4.11	5	0.04	8.21	10	7.71	7	-0.50
Denmark	5.66	11	5.60	6	-0.06	5.22	5	4.92	3	-0.30
Spain	6.37	12	12.56	11	6.19	9.10	12	13.88	13	4.78
Estonia	17.28	18	15.36	14	-1.92	20.64	17	23.07	20	2.43
Finland	4.89	10	11.41	9	6.52	5.65	6	7.22	6	1.57
France	4.15	9	22.00	21	17.85	7.73	9	24.54	21	16.81
Croatia	18.72	19	18.41	16	-0.31	21.01	18	16.60	15	-4.41
Hungary	16.95	17	21.00	20	4.05	24.11	20	19.56	18	-4.55
Iceland	0.01	3	104.21	25	104.20	-3.12	1	18.41	17	21.53
Italy	6.93	13	9.54	8	2.61	10.97	13	8.79	10	-2.18
Lithuania	25.84	22	18.65	17	-7.19	23.63	19	16.55	14	-7.08
Luxembourg	2.01	6	34.53	23	32.52	3.72	3	48.27	24	44.55
Latvia	26.06	23	13.35	12	-12.71	26.48	21	88.09	25	61.61
Netherlands	9.15	15	11.45	10	2.30	13.87	15	12.91	11	-0.96
Norway	3.62	7	3.33	4	-0.29	2.48	2	-0.60	1	-3.08
Poland	24.25	21	20.16	19	-4.09	27.97	22	20.05	19	-7.92
Portugal	11.11	16	19.19	18	8.08	11.75	14	13.76	12	2.01
Slovakia	37.17	24	23.81	22	-13.36	38.03	24	27.53	22	-10.50
Slovenia	7.19	14	16.99	15	9.80	16.31	16	3.61	2	-12.70
Sweden	0.97	4	1.44	2	0.47	4.55	4	6.86	4	2.31

Supplementary Table 5: P-scores, standardized P-scores, the difference between P-score and the standardized P-score (ΔP-score) and their respective rank (#) in 2021 for men and women for all included European countries. Standardization was done using a uniform density. Source: World Population Prospects (2024), Short Term Mortality Fluctuation data series (2024)

Country	Female					Male				
	P-score	#	P-score$_{st.}$	#	ΔP-score	P-score	#	P-score$_{st.}$	#	ΔP-score
Austria	-4.06	1	7.35	8	11.41	8.40	9	10.62	14	2.22
Belgium	3.15	3	6.88	7	3.73	6.72	3	5.45	2	-1.27
Bulgaria	14.41	24	8.45	11	-5.96	16.16	25	7.65	6	-8.51
Switzerland	5.92	8	9.99	14	4.07	8.96	11	12.05	16	3.09
Czechia	6.03	9	5.27	3	-0.76	7.65	6	6.19	3	-1.46
Germany	7.46	12	8.55	12	1.09	10.36	17	10.19	13	-0.17
Denmark	9.93	15	8.61	13	-1.32	8.89	10	7.57	5	-1.32
Spain	10.59	17	17.64	21	7.05	10.34	16	23.48	22	13.14
Estonia	7.01	10	16.83	18	9.82	11.85	20	18.86	21	7.01
Finland	15.14	25	13.79	17	-1.35	14.75	24	11.95	15	-2.80
France	7.37	11	35.34	24	27.97	9.01	12	26.96	23	17.95
Croatia	8.68	14	5.34	4	-3.34	6.76	4	15.64	18	8.88
Hungary	3.85	4	6.47	6	2.62	8.02	8	7.99	7	-0.03
Iceland	14.28	23	80.49	25	66.21	7.50	5	8.91	8	1.41
Italy	8.27	13	5.72	5	-2.55	9.28	13	9.43	11	0.15
Lithuania	13.54	22	4.61	2	-8.93	10.15	15	-2.47	1	-12.62
Luxembourg	0.76	2	25.00	22	24.24	-0.04	1	54.67	24	54.71
Latvia	11.38	20	10.30	15	-1.08	14.60	23	154.85	25	140.25
Netherlands	10.13	16	17.15	19	7.02	10.49	18	17.69	20	7.20
Norway	11.21	19	8.04	10	-3.17	13.01	22	13.54	17	0.53
Poland	5.78	6	10.46	16	4.68	8.00	7	9.52	12	1.52
Portugal	12.53	21	25.63	23	13.10	10.60	19	15.82	19	5.22
Slovakia	10.77	18	7.98	9	-2.79	11.97	21	9.13	10	-2.84
Slovenia	5.83	7	17.64	20	11.81	9.28	14	7.30	4	-1.98
Sweden	4.48	5	1.37	1	-3.11	6.20	2	9.02	9	2.82

Supplementary Table 6: P-scores, standardized P-scores, the difference between P-score and the standardized P-score (ΔP-score) and their respective rank (#) in 2022 for men and women for all included European countries. Standardization was done using a uniform density. Source: World Population Prospects (2024), Short Term Mortality Fluctuation data series (2024)

Country	Female					Male				
	P-score	#	P-score$_{st.}$	#	ΔP-score	P-score	#	P-score$_{st.}$	#	ΔP-score
Austria	-10.95	1	3.23	10	14.18	4.63	15	11.24	13	6.61
Belgium	-3.52	5	1.56	8	5.08	2.14	10	4.89	8	2.75
Bulgaria	-2.02	9	-7.49	1	-5.47	-0.62	4	3.23	5	3.85
Switzerland	0.92	15	8.62	16	7.70	3.39	12	13.07	14	9.68
Czechia	-2.77	7	-2.12	4	0.65	0.45	7	0.14	3	-0.31
Germany	2.23	18	4.84	13	2.61	5.44	18	8.74	11	3.30
Denmark	6.16	22	4.70	11	-1.46	6.94	22	3.09	4	-3.85
Spain	2.92	19	17.29	21	14.37	3.50	13	16.27	17	12.77
Estonia	-3.28	6	14.51	19	17.79	3.87	14	16.21	16	12.34
Finland	9.79	25	16.46	20	6.67	12.45	25	16.95	18	4.50
France	1.23	16	28.53	23	27.30	3.07	11	24.55	23	21.48
Croatia	-4.11	3	-0.65	6	3.46	-3.22	3	17.95	20	21.17
Hungary	-3.91	4	-0.15	7	3.76	1.33	8	3.52	6	2.19
Iceland	2.05	17	107.18	25	105.13	5.11	17	29.72	24	24.61
Italy	-0.97	11	6.94	14	7.91	1.48	9	6.71	9	5.23
Lithuania	-0.88	12	-1.86	5	-0.98	0.25	6	-1.73	1	-1.98
Luxembourg	0.48	14	29.30	24	28.82	-4.67	1	15.98	15	20.65
Latvia	-0.46	13	-2.56	3	-2.10	5.71	19	95.37	25	89.66
Netherlands	9.03	24	11.73	17	2.70	10.09	24	18.95	21	8.86
Norway	6.75	23	13.44	18	6.69	8.05	23	17.45	19	9.40
Poland	-5.90	2	2.39	9	8.29	-3.47	2	-0.79	2	2.68
Portugal	5.60	21	24.29	22	18.69	6.00	20	20.69	22	14.69
Slovakia	-1.89	10	-5.54	2	-3.65	0.09	5	3.95	7	3.86
Slovenia	-2.70	8	8.35	15	11.05	6.26	21	8.59	10	2.33
Sweden	2.95	20	4.70	12	1.75	5.05	16	9.40	12	4.35

Supplementary Table 7: P-scores, standardized P-scores, the difference between P-score and the standardized P-score (ΔP-score) and their respective rank (#) in 2023 for men and women for all included European countries. Standardization was done using a uniform density. Source: World Population Prospects (2024), Short Term Mortality Fluctuation data series (2024)
